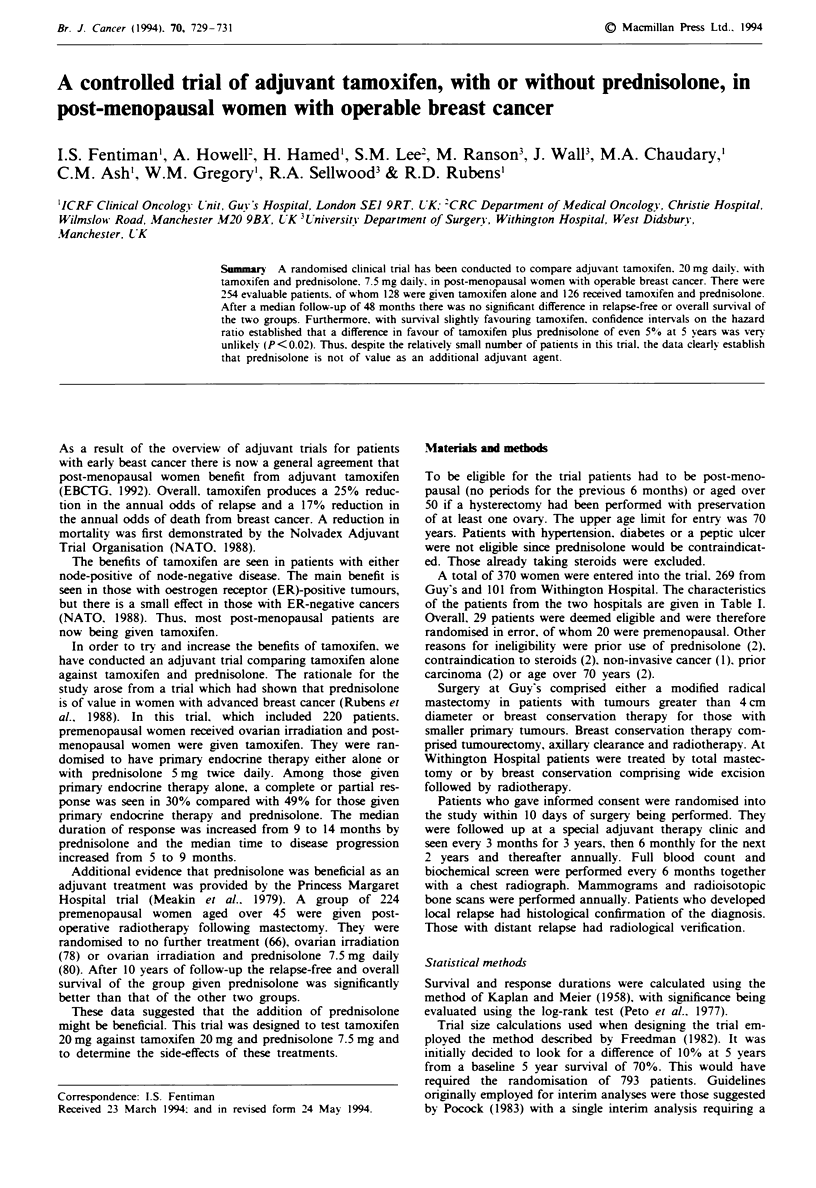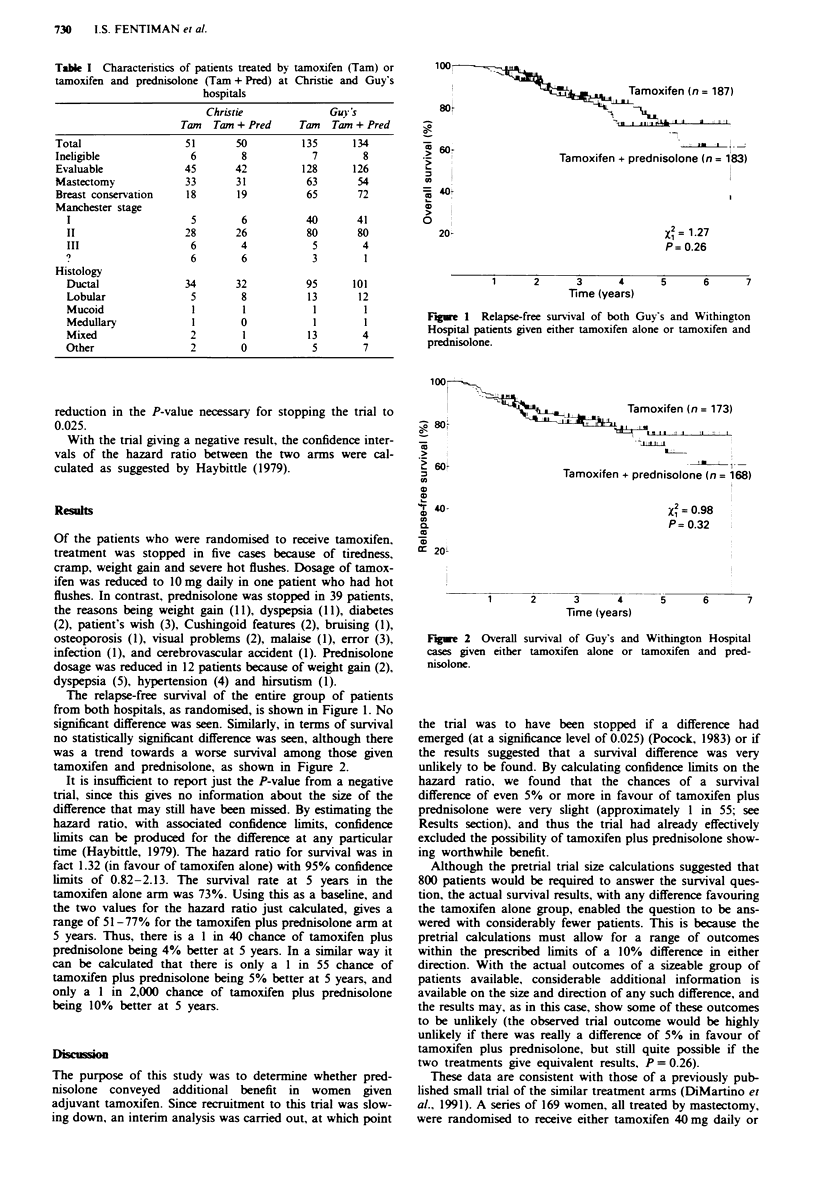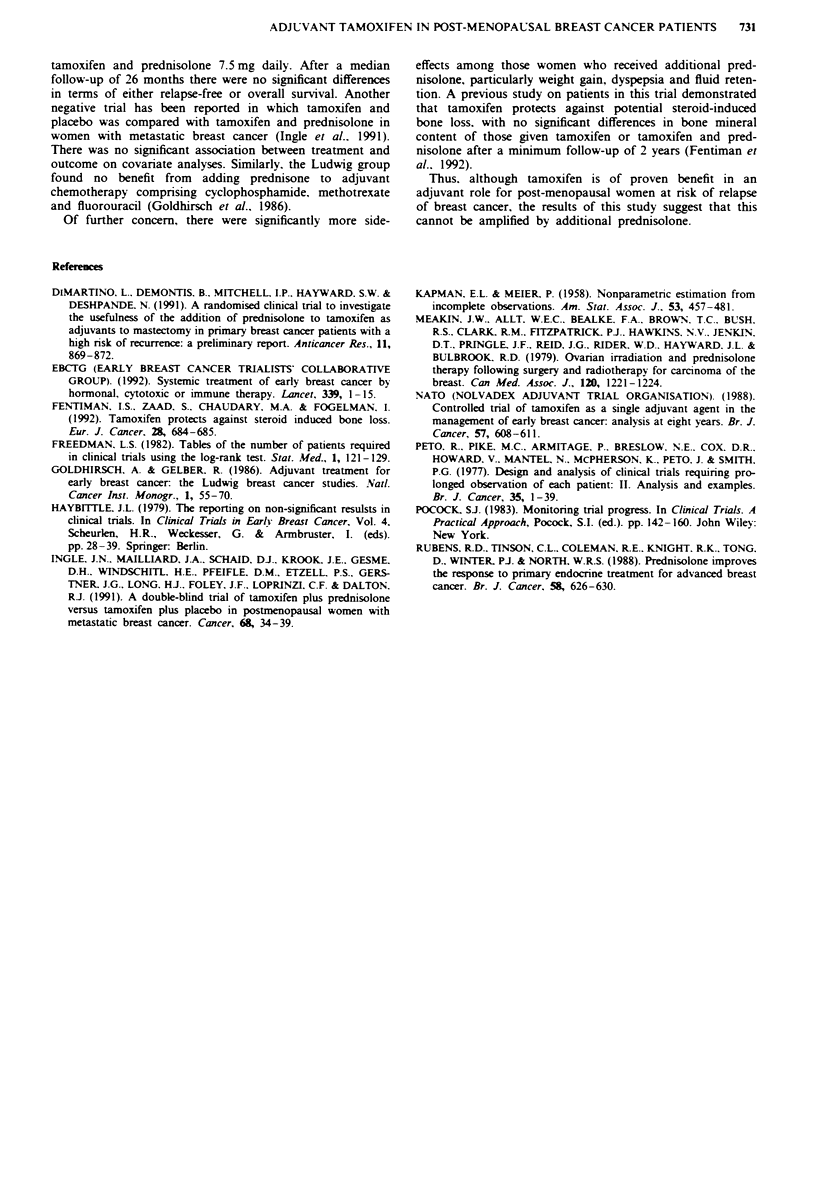# A controlled trial of adjuvant tamoxifen, with or without prednisolone, in post-menopausal women with operable breast cancer.

**DOI:** 10.1038/bjc.1994.384

**Published:** 1994-10

**Authors:** I. S. Fentiman, A. Howell, H. Hamed, S. M. Lee, M. Ranson, J. Wall, M. A. Chaudary, C. M. Ash, W. M. Gregory, R. A. Sellwood

**Affiliations:** ICRF Clinical Oncology Unit, Guy's Hospital, London, UK.

## Abstract

A randomised clinical trial has been conducted to compare adjuvant tamoxifen, 20 mg daily, with tamoxifen and prednisolone, 7.5 mg daily, in post-menopausal women with operable breast cancer. There were 254 evaluable patients, of whom 128 were given tamoxifen alone and 126 received tamoxifen and prednisolone. After a median follow-up of 48 months there was no significant difference in relapse-free or overall survival of the two groups. Furthermore, with survival slightly favouring tamoxifen, confidence intervals on the hazard ratio established that a difference in favour of tamoxifen plus prednisolone of even 5% at 5 years was very unlikely (P < 0.02). Thus, despite the relatively small number of patients in this trial, the data clearly establish that prednisolone is not of value as an additional adjuvant agent.


Br. J. Cancer (1994), 70, 729-731 © Macmillan Press Ltd., 1994

### A controlled trial of adjuvant tamoxifen, with or without prednisolone, in post-menopausal women with operable breast cancer

I.S. Fentiman[1], A. Howell[2], H. Hamed[1], S.M. Lee[2], M. Ranson[3], J. Wall[3], M.A. Chaudary,[1] C.M. Ash[1], W.M. Gregory[1], R.A. Sellwood[3] & R.D. Rubens[1]

[1]ICRF Clinical Oncology Unit, Guy's Hospital, London SE1 9RT, UK; [2]CRC Department of Medical Oncology, Christie Hospital, Wilmslow Road, Manchester M20 9BX, UK [3]University Department of Surgery, Withington Hospital, West Didsbury, Manchester, UK

**Summary** A randomised clinical trial has been conducted to compare adjuvant tamoxifen, 20 mg daily, with tamoxifen and prednisolone, 7.5 mg daily, in post-menopausal women with operable breast cancer. There were 254 evaluable patients, of whom 128 were given tamoxifen alone and 126 received tamoxifen and prednisolone. After a median follow-up of 48 months there was no significant difference in relapse-free or overall survival of the two groups. Furthermore, with survival slightly favouring tamoxifen, confidence intervals on the hazard ratio established that a difference in favour of tamoxifen plus prednisolone of even 5% at 5 years was very unlikely ($P<0.02$). Thus, despite the relatively small number of patients in this trial, the data clearly establish that prednisolone is not of value as an additional adjuvant agent.

As a result of the overview of adjuvant trials for patients with early beast cancer there is now a general agreement that post-menopausal women benefit from adjuvant tamoxifen (EBCTG, 1992). Overall, tamoxifen produces a 25% reduction in the annual odds of relapse and a 17% reduction in the annual odds of death from breast cancer. A reduction in mortality was first demonstrated by the Nolvadex Adjuvant Trial Organisation (NATO, 1988).

The benefits of tamoxifen are seen in patients with either node-positive of node-negative disease. The main benefit is seen in those with oestrogen receptor (ER)-positive tumours, but there is a small effect in those with ER-negative cancers (NATO, 1988). Thus, most post-menopausal patients are now being given tamoxifen.

In order to try and increase the benefits of tamoxifen, we have conducted an adjuvant trial comparing tamoxifen alone against tamoxifen and prednisolone. The rationale for the study arose from a trial which had shown that prednisolone is of value in women with advanced breast cancer (Rubens *et al.*, 1988). In this trial, which included 220 patients, premenopausal women received ovarian irradiation and post-menopausal women were given tamoxifen. They were randomised to have primary endocrine therapy either alone or with prednisolone 5 mg twice daily. Among those given primary endocrine therapy alone, a complete or partial response was seen in 30% compared with 49% for those given primary endocrine therapy and prednisolone. The median duration of response was increased from 9 to 14 months by prednisolone and the median time to disease progression increased from 5 to 9 months.

Additional evidence that prednisolone was beneficial as an adjuvant treatment was provided by the Princess Margaret Hospital trial (Meakin *et al.*, 1979). A group of 224 premenopausal women aged over 45 were given post-operative radiotherapy following mastectomy. They were randomised to no further treatment (66), ovarian irradiation (78) or ovarian irradiation and prednisolone 7.5 mg daily (80). After 10 years of follow-up the relapse-free and overall survival of the group given prednisolone was significantly better than that of the other two groups.

These data suggested that the addition of prednisolone might be beneficial. This trial was designed to test tamoxifen 20 mg against tamoxifen 20 mg and prednisolone 7.5 mg and to determine the side-effects of these treatments.

Correspondence: I.S. Fentiman
Received 23 March 1994; and in revised form 24 May 1994.

### Materials and methods

To be eligible for the trial patients had to be post-menopausal (no periods for the previous 6 months) or aged over 50 if a hysterectomy had been performed with preservation of at least one ovary. The upper age limit for entry was 70 years. Patients with hypertension, diabetes or a peptic ulcer were not eligible since prednisolone would be contraindicated. Those already taking steroids were excluded.

A total of 370 women were entered into the trial, 269 from Guy's and 101 from Withington Hospital. The characteristics of the patients from the two hospitals are given in Table I. Overall, 29 patients were deemed eligible and were therefore randomised in error, of whom 20 were premenopausal. Other reasons for ineligibility were prior use of prednisolone (2), contraindication to steroids (2), non-invasive cancer (1), prior carcinoma (2) or age over 70 years (2).

Surgery at Guy's comprised either a modified radical mastectomy in patients with tumours greater than 4 cm diameter or breast conservation therapy for those with smaller primary tumours. Breast conservation therapy comprised tumourectomy, axillary clearance and radiotherapy. At Withington Hospital patients were treated by total mastectomy or by breast conservation comprising wide excision followed by radiotherapy.

Patients who gave informed consent were randomised into the study within 10 days of surgery being performed. They were followed up at a special adjuvant therapy clinic and seen every 3 months for 3 years, then 6 monthly for the next 2 years and thereafter annually. Full blood count and biochemical screen were performed every 6 months together with a chest radiograph. Mammograms and radioisotopic bone scans were performed annually. Patients who developed local relapse had histological confirmation of the diagnosis. Those with distant relapse had radiological verification.

### Statistical methods

Survival and response durations were calculated using the method of Kaplan and Meier (1958), with significance being evaluated using the log-rank test (Peto *et al.*, 1977).

Trial size calculations used when designing the trial employed the method described by Freedman (1982). It was initially decided to look for a difference of 10% at 5 years from a baseline 5 year survival of 70%. This would have required the randomisation of 793 patients. Guidelines originally employed for interim analyses were those suggested by Pocock (1983) with a single interim analysis requiring a

**Table I** Characteristics of patients treated by tamoxifen (Tam) or tamoxifen and prednisolone (Tam + Pred) at Christie and Guy's hospitals

| | *Christie* | | *Guy's* | |
|---|---|---|---|---|
| | *Tam* | *Tam + Pred* | *Tam* | *Tam + Pred* |
| Total | 51 | 50 | 135 | 134 |
| Ineligible | 6 | 8 | 7 | 8 |
| Evaluable | 45 | 42 | 128 | 126 |
| Mastectomy | 33 | 31 | 63 | 54 |
| Breast conservation | 18 | 19 | 65 | 72 |
| Manchester stage | | | | |
| I | 5 | 6 | 40 | 41 |
| II | 28 | 26 | 80 | 80 |
| III | 6 | 4 | 5 | 4 |
| ? | 6 | 6 | 3 | 1 |
| Histology | | | | |
| Ductal | 34 | 32 | 95 | 101 |
| Lobular | 5 | 8 | 13 | 12 |
| Mucoid | 1 | 1 | 1 | 1 |
| Medullary | 1 | 0 | 1 | 1 |
| Mixed | 2 | 1 | 13 | 4 |
| Other | 2 | 0 | 5 | 7 |

reduction in the $P$-value necessary for stopping the trial to 0.025.

With the trial giving a negative result, the confidence intervals of the hazard ratio between the two arms were calculated as suggested by Haybittle (1979).

### Results

Of the patients who were randomised to receive tamoxifen, treatment was stopped in five cases because of tiredness, cramp, weight gain and severe hot flushes. Dosage of tamoxifen was reduced to 10 mg daily in one patient who had hot flushes. In contrast, prednisolone was stopped in 39 patients, the reasons being weight gain (11), dyspepsia (11), diabetes (2), patient's wish (3), Cushingoid features (2), bruising (1), osteoporosis (1), visual problems (2), malaise (1), error (3), infection (1), and cerebrovascular accident (1). Prednisolone dosage was reduced in 12 patients because of weight gain (2), dyspepsia (5), hypertension (4) and hirsutism (1).

The relapse-free survival of the entire group of patients from both hospitals, as randomised, is shown in Figure 1. No significant difference was seen. Similarly, in terms of survival no statistically significant difference was seen, although there was a trend towards a worse survival among those given tamoxifen and prednisolone, as shown in Figure 2.

It is insufficient to report just the $P$-value from a negative trial, since this gives no information about the size of the difference that may still have been missed. By estimating the hazard ratio, with associated confidence limits, confidence limits can be produced for the difference at any particular time (Haybittle, 1979). The hazard ratio for survival was in fact 1.32 (in favour of tamoxifen alone) with 95% confidence limits of 0.82–2.13. The survival rate at 5 years in the tamoxifen alone arm was 73%. Using this as a baseline, and the two values for the hazard ratio just calculated, gives a range of 51–77% for the tamoxifen plus prednisolone arm at 5 years. Thus, there is a 1 in 40 chance of tamoxifen plus prednisolone being 4% better at 5 years. In a similar way it can be calculated that there is only a 1 in 55 chance of tamoxifen plus prednisolone being 5% better at 5 years, and only a 1 in 2,000 chance of tamoxifen plus prednisolone being 10% better at 5 years.

### Discussion

The purpose of this study was to determine whether prednisolone conveyed additional benefit in women given adjuvant tamoxifen. Since recruitment to this trial was slowing down, an interim analysis was carried out, at which point

**Figure 1** Relapse-free survival of both Guy's and Withington Hospital patients given either tamoxifen alone or tamoxifen and prednisolone.

**Figure 2** Overall survival of Guy's and Withington Hospital cases given either tamoxifen alone or tamoxifen and prednisolone.

the trial was to have been stopped if a difference had emerged (at a significance level of 0.025) (Pocock, 1983) or if the results suggested that a survival difference was very unlikely to be found. By calculating confidence limits on the hazard ratio, we found that the chances of a survival difference of even 5% or more in favour of tamoxifen plus prednisolone were very slight (approximately 1 in 55; see Results section), and thus the trial had already effectively excluded the possibility of tamoxifen plus prednisolone showing worthwhile benefit.

Although the pretrial trial size calculations suggested that 800 patients would be required to answer the survival question, the actual survival results, with any difference favouring the tamoxifen alone group, enabled the question to be answered with considerably fewer patients. This is because the pretrial calculations must allow for a range of outcomes within the prescribed limits of a 10% difference in either direction. With the actual outcomes of a sizeable group of patients available, considerable additional information is available on the size and direction of any such difference, and the results may, as in this case, show some of these outcomes to be unlikely (the observed trial outcome would be highly unlikely if there was really a difference of 5% in favour of tamoxifen plus prednisolone, but still quite possible if the two treatments give equivalent results, $P = 0.26$).

These data are consistent with those of a previously published small trial of the similar treatment arms (DiMartino *et al.*, 1991). A series of 169 women, all treated by mastectomy, were randomised to receive either tamoxifen 40 mg daily or tamoxifen and prednisolone 7.5 mg daily. After a median follow-up of 26 months there were no significant differences in terms of either relapse-free or overall survival. Another negative trial has been reported in which tamoxifen and placebo was compared with tamoxifen and prednisolone in women with metastatic breast cancer (Ingle *et al*., 1991). There was no significant association between treatment and outcome on covariate analyses. Similarly, the Ludwig group found no benefit from adding prednisone to adjuvant chemotherapy comprising cyclophosphamide, methotrexate and fluorouracil (Goldhirsch *et al*., 1986).

Of further concern, there were significantly more side-effects among those women who received additional prednisolone, particularly weight gain, dyspepsia and fluid retention. A previous study on patients in this trial demonstrated that tamoxifen protects against potential steroid-induced bone loss, with no significant differences in bone mineral content of those given tamoxifen or tamoxifen and prednisolone after a minimum follow-up of 2 years (Fentiman *et al*., 1992).

Thus, although tamoxifen is of proven benefit in an adjuvant role for post-menopausal women at risk of relapse of breast cancer, the results of this study suggest that this cannot be amplified by additional prednisolone.

**References**

DIMARTINO, L., DEMONTIS, B., MITCHELL, I.P., HAYWARD, S.W. & DESHPANDE, N. (1991). A randomised clinical trial to investigate the usefulness of the addition of prednisolone to tamoxifen as adjuvants to mastectomy in primary breast cancer patients with a high risk of recurrence: a preliminary report. *Anticancer Res*., **11**, 869-872.

EBCTG (EARLY BREAST CANCER TRIALISTS' COLLABORATIVE GROUP). (1992). Systemic treatment of early breast cancer by hormonal, cytotoxic or immune therapy. *Lancet*, **339**, 1-15.

FENTIMAN, I.S., ZAAD, S., CHAUDARY, M.A. & FOGELMAN, I. (1992). Tamoxifen protects against steroid induced bone loss. *Eur. J. Cancer*, **28**, 684-685.

FREEDMAN, L.S. (1982). Tables of the number of patients required in clinical trials using the log-rank test. *Stat. Med*., **1**, 121-129.

GOLDHIRSCH, A. & GELBER, R. (1986). Adjuvant treatment for early breast cancer: the Ludwig breast cancer studies. *Natl. Cancer Inst. Monogr*., **1**, 55-70.

HAYBITTLE, J.L. (1979). The reporting on non-significant resulsts in clinical trials. In *Clinical Trials in Early Breast Cancer*, Vol. 4, Scheurlen, H.R., Weckesser, G. & Armbruster, I. (eds). pp. 28-39. Springer: Berlin.

INGLE, J.N., MAILLIARD, J.A., SCHAID, D.J., KROOK, J.E., GESME, D.H., WINDSCHITL, H.E., PFEIFLE, D.M., ETZELL, P.S., GERSTNER, J.G., LONG, H.J., FOLEY, J.F., LOPRINZI, C.F. & DALTON, R.J. (1991). A double-blind trial of tamoxifen plus prednisolone versus tamoxifen plus placebo in postmenopausal women with metastatic breast cancer. *Cancer*, **68**, 34-39.

KAPMAN, E.L. & MEIER, P. (1958). Nonparametric estimation from incomplete observations. *Am. Stat. Assoc. J*., **53**, 457-481.

MEAKIN, J.W., ALLT, W.E.C., BEALKE, F.A., BROWN, T.C., BUSH, R.S., CLARK, R.M., FITZPATRICK, P.J., HAWKINS, N.V., JENKIN, D.T., PRINGLE, J.F., REID, J.G., RIDER, W.D., HAYWARD, J.L. & BULBROOK, R.D. (1979). Ovarian irradiation and prednisolone therapy following surgery and radiotherapy for carcinoma of the breast. *Can Med. Assoc. J*., **120**, 1221-1224.

NATO (NOLVADEX ADJUVANT TRIAL ORGANISATION). (1988). Controlled trial of tamoxifen as a single adjuvant agent in the management of early breast cancer: analysis at eight years. *Br. J. Cancer*, **57**, 608-611.

PETO, R., PIKE, M.C., ARMITAGE, P., BRESLOW, N.E., COX, D.R., HOWARD, V., MANTEL, N., MCPHERSON, K., PETO, J. & SMITH, P.G. (1977). Design and analysis of clinical trials requiring prolonged observation of each patient: II. Analysis and examples. *Br. J. Cancer*, **35**, 1-39.

POCOCK, S.J. (1983). Monitoring trial progress. In *Clinical Trials. A Practical Approach*, Pocock, S.I. (ed.). pp. 142-160. John Wiley: New York.

RUBENS, R.D., TINSON, C.L., COLEMAN, R.E., KNIGHT, R.K., TONG, D., WINTER, P.J. & NORTH, W.R.S. (1988). Prednisolone improves the response to primary endocrine treatment for advanced breast cancer. *Br. J. Cancer*, **58**, 626-630.